# The Role of Prostate-Specific Membrane Antigen (PSMA) PET-CT in Characterizing Suspected Synchronous Rectal Lesions in a Case of High-Grade Prostatic Adenocarcinoma

**DOI:** 10.7759/cureus.91575

**Published:** 2025-09-03

**Authors:** Gowri Sankar, Swarnava Tarafdar, Anindya Halder, Rama Saha, Santosh Tummidi

**Affiliations:** 1 Nuclear Medicine, All India Institute of Medical Sciences, Kalyani, IND; 2 Radiology, All India Institute of Medical Sciences, Kalyani, IND; 3 General Surgery, All India Institute of Medical Sciences, Kalyani, IND; 4 Pathology and Laboratory Medicine, All India Institute of Medical Sciences, Kalyani, IND

**Keywords:** ga-68 psma pet-ct, high-grade prostate adenocarcinoma, primary score, promise v2 criteria, synchronous rectal lesions

## Abstract

We report a case of a 65-year-old man who presented with urinary voiding problems and inadequate bowel evacuation. On digital rectal examination (DRE), he was found to have a hard mass in the prostate. Colonoscopy revealed a rectal proliferative growth, which was also suspicious for malignancy. Initially, a clinical diagnosis of prostate carcinoma with rectal involvement was suspected.

The patient then underwent radiological evaluation in the form of computed tomography (CT) and magnetic resonance imaging (MRI) scans, which revealed two separate lesions in the prostate and rectum with maintained fat planes along with pelvic and abdominal lymphadenopathy and skeletal metastases. Hence, a radiological diagnosis of prostatic cancer with synchronous rectal primary was made. Further histopathological evaluation revealed high-grade prostatic adenocarcinoma. However, rectal biopsy revealed an inflammatory lesion in the rectum, even in repeated biopsies, in spite of high clinical and radiological suspicion of synchronous rectal primary. Later, gallium-68-labeled PSMA positron emission tomography-computed tomography (Ga-68 PSMA PET-CT) scan revealed the prostatic lesion with low to intermediate expression of PSMA, along with multiple metastatic pelvic and abdominal lymph nodes and extensive skeletal metastases with intermediate to high PSMA expression. However, the rectal lesion showed low PSMA expression.

This case highlights the fact that PSMA expression in suspicious rectal lesions can help in labeling them as benign or malignant. In this patient, it aligned with repeated biopsy results, which showed benign proctitis, despite high clinical and radiological suspicion of malignancy.

## Introduction

Prostate cancer is the second most common malignancy affecting men worldwide, with the most common pathological subtype being acinar adenocarcinoma [1]. Adenocarcinoma prostate cells express prostate-specific membrane antigen (PSMA) at a higher concentration than the rest of the prostate gland. This PSMA expression also increases with an increase in tumor grade of the lesion, as well as in metastatic disease. Gallium-68-labeled PSMA positron emission tomography-computed tomography (Ga-68 PSMA PET-CT) scan is used for staging intermediate- to high-grade prostate cancer, restaging, and before starting radionuclide therapy in metastatic castrate-resistant prostate cancer [2].

PSMA expression score obtained from Ga-68 PSMA PET-CT scan increases with an increase in Gleason's score, grade group of the tumor, and in extensive metastatic disease, and thus is included in the Prostate Cancer Molecular Imaging Standardized Evaluation (PROMISE) V2 criteria [3]. The PROMISE V2 criteria include an updated molecular imaging TNM (miTNM) whole-body staging system and PSMA expression score for all morphologically significant lesions. PSMA expression score is a 4-point score based on visual assessment of PSMA uptake in morphologically significant lesions: score 0 depicts an uptake equal to or lower than blood pool activity and signifies no PSMA expression; score 1 means an uptake higher than blood pool activity but equal to or less than liver and signifies low PSMA expression; score 2 means an uptake more than the uptake seen in liver but equal to or less than the uptake seen in parotid glands, and it signifies intermediate PSMA expression; and the highest score 3 means an uptake higher than parotid gland uptake, and it signifies a high PSMA expression. This expression score is used on a per-lesion basis and is required to determine whether that patient is eligible for PSMA radioligand therapy [3].

The PROMISE V2 criteria also incorporate the PRIMARY score, which is a 5-point scoring system, ranging from 1 to 5, for the assessment of intraprostatic lesions. It is a combination of the intensity of PSMA uptake in intraprostatic lesions and their location and uptake pattern. Only the most clinically significant lesion will be used for assigning the PRIMARY score [3].

Although it is labeled as prostate-specific, this membrane antigen expression is not limited only to prostate cancer cells, as the name suggests. PSMA is also expressed in certain non-prostatic malignancies and benign conditions. PSMA is a transmembrane glycoprotein encoded by the *FOLH1* gene, and it is also expressed in various non-prostatic malignancies, predominantly in the neovasculature, along the endothelium [4].

## Case presentation

A 65-year-old man presented with complaints of decreased urine output, difficulty in urination, incomplete evacuation of bowel, and generalized body pain. On digital rectal examination (DRE), a hard mass was felt at the tip of the index finger. Colonoscopy revealed a suspicious rectal proliferative growth, beyond which hard stool accumulation was present.

Contrast-enhanced computed tomography (CECT) scan revealed asymmetric heterogeneously enhancing circumferential wall thickening of the rectosigmoid junction, and proximal and mid rectum, along with pelvic and abdominal lymphadenopathy compressing bilateral distal ureters, leading to bilateral proximal hydroureteronephrosis. The prostate appeared enlarged with heterogeneous enhancement and irregular margins involving the base of the urinary bladder and infiltrating seminal vesicles. Multiple mixed lytic and sclerotic skeletal lesions were also noted involving visualized vertebrae, pelvic bones, and bilateral femora. Fat planes between the prostate and rectal lesion were maintained, and the prostate lesion did not directly invade the rectum.

**Figure 1:**
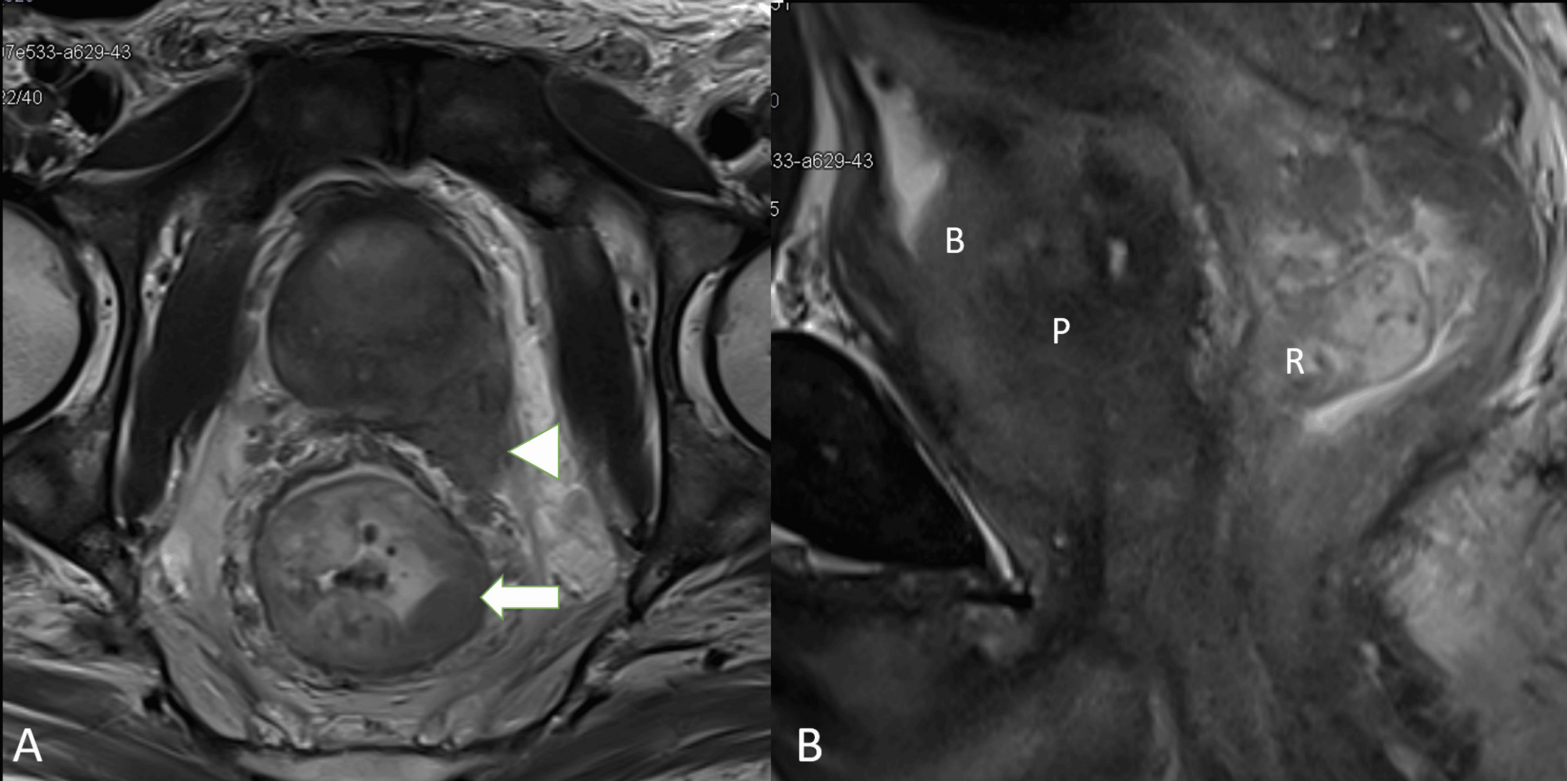
T2W MRI T2W MRI axial and sagittal views (A and B) show irregular circumferential rectal thickening (arrow) with a few suspicious mesorectal lymph nodes. Prostatic mass invades the left seminal vesicle, neurovascular bundle, and urinary bladder (arrowhead), with no direct invasion into the rectum. T2W MRI: T2-weighted magnetic resonance imaging, P: prostate, B: urinary bladder, R: rectum

Contrast-enhanced magnetic resonance imaging (CE-MRI) confirmed CECT findings, with maintained fat planes between prostatic and rectal lesions (Figure 1). Hence, a diagnosis of prostate carcinoma with suspected synchronous rectal primary along with metastatic lymphadenopathy and skeletal metastases was made based on clinical and radiological findings.

**Figure 2:**
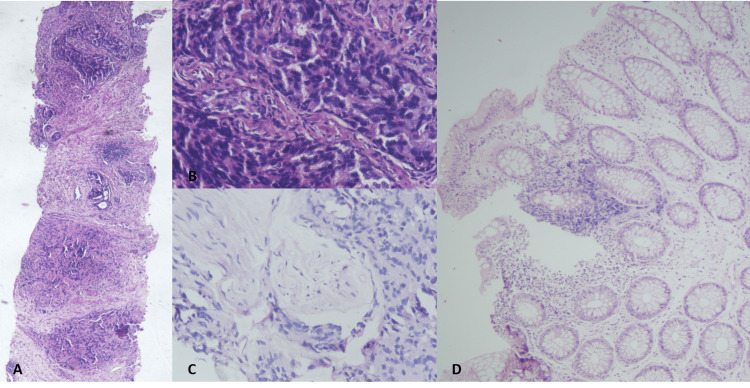
Histopathological evaluation of prostatic and rectal biopsy Histopathological evaluation of TRUS-guided prostatic biopsy with H&E stain showing tumor cells arranged in sheets and cribriform glandular pattern with cells showing pleomorphic nuclei, high N:C ratio, and prominent nucleoli characteristic of acinar adenocarcinoma of the prostate with perineural invasion under 4× (A) and 40× magnifications (B and C), with a Gleason score of 5+4, grade group 5. Rectal biopsy shows edematous lamina propria with mild lymphocytic infiltration and focal lymphoid aggregates with no evidence of malignancy or dysplasia (H&E stain, 10× magnification) (D). TRUS: transrectal ultrasound, H&E: hematoxylin and eosin

The patient then underwent rectal biopsy, which showed edematous lamina propria with lymphocytic infiltrates and focal lymphoid aggregates. No evidence of dysplasia or malignancy was seen in the sections examined, leading to a pathological diagnosis of chronic proctitis. Since clinical and radiological suspicion was very high for a synchronous rectal primary and to avoid possible false-negative biopsy risk, a repeat biopsy was done, which confirmed the initial biopsy findings (Figure 2). Transrectal ultrasound (TRUS)-guided prostatic biopsy revealed acinar adenocarcinoma, conventional type in both lobes with modified Gleason score of 9 (5+4) and International Society of Urological Pathology (ISUP) grade group 5, along with perineural invasion (Figure 2).

The serum prostate-specific antigen (PSA) and creatinine levels of the patient were elevated, measuring 413.82 ng/mL (normal range: 0-4 ng/mL) and 3.41 mg/dL (normal range: 0.6-1.2 mg/dL), respectively. The elevated serum creatinine levels were due to obstructive uropathy, caused by prostatic malignancy. The likely cause of anemia in this patient was proctitis. His serum carcinoembryonic antigen (CEA) levels were within normal limits (Table 1).

**Table 1:** Blood test results Hb: hemoglobin, PSA: prostate-specific antigen, ALP: alkaline phosphatase, CEA: carcinoembryonic antigen

Blood test	Patient value	Normal range
Hb	8.1 g/dL (↓)	13.5-17.5 g/dL
Serum creatinine	3.41 mg/dL (↑)	0.6-1.2 mg/dL
Serum PSA	413.82 ng/mL (↑↑)	0-4 ng/mL
Serum ALP	105 U/L	24-147 U/L
Serum CEA	3.05 ng/mL	<5 ng/mL

**Figure 3:**
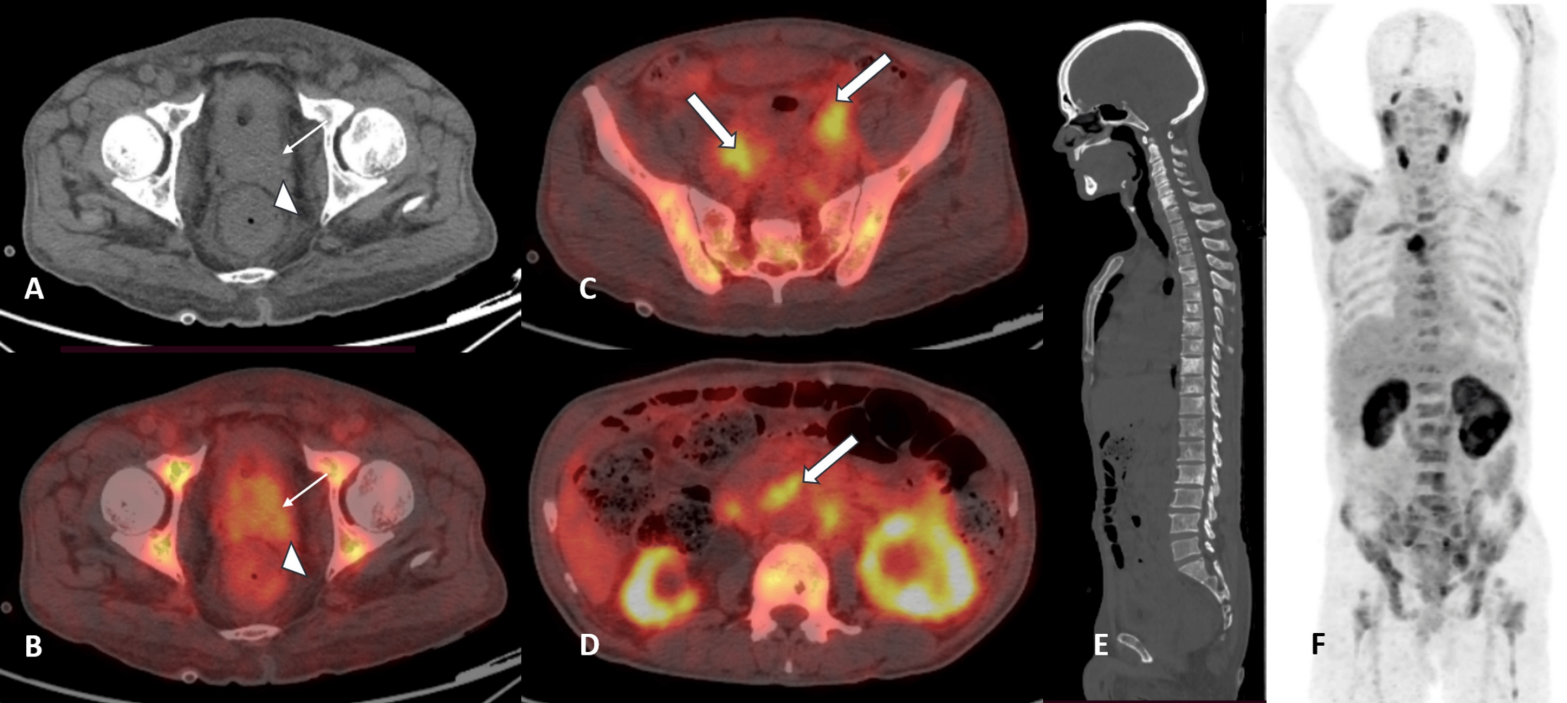
Ga-68 PSMA PET-CT images Trans-axial section views of non-contrast CT (A) and fused Ga-68 PSMA PET-CT images (B-D) showing prostatic primary lesion infiltrating the bladder and urinary bladder wall with low to intermediate PSMA expression (thin arrows in A and B). Also, circumferential mural thickening was noted in the rectum with low-grade PSMA expression (arrowheads in A and B). Fat planes between prostatic and rectal lesions are maintained. Multiple pelvic and retroperitoneal lymph nodes were noted with intermediate- to high-grade PSMA expression (thick arrows in C and D). Maximum intensity projection images (F) showing multiple intermediate to high PSMA-expressing skeletal lesions, with the highest PSMA expression in the sternal lesion. Sagittal section of non-contrast CT images (E) showing multiple mixed lytic and sclerotic skeletal lesions (predominantly sclerotic) in the visualized axial skeleton. Ga-68 PSMA PET-CT: gallium-68-labeled PSMA positron emission tomography-computed tomography

The patient then underwent Ga-68 PSMA PET-CT scan without intravenous contrast, which revealed an enlarged prostate with diffuse low to intermediate PSMA expression (miPSMA expression score of 1-2, with the highest score of 2), infiltrating the urinary bladder and bilateral seminal vesicles. Rectal lesion uptake was visually similar to that of the prostatic lesion (miPSMA expression score of 1). The maximum standardized uptake values (SUV max) were 3.2 and 3.8 for rectal and prostatic lesions, respectively, with reference liver SUV max in the right lobe measuring 3.2. Multiple bilateral pelvic lymphadenopathies, including peri-rectal, presacral, and retroperitoneal, were noted with intermediate to high PSMA expression (miPSMA expression score of 2-3, with the highest score of 3), which were compressing bilateral distal ureters, leading to proximal mild to moderate hydroureteronephrosis. Multiple mixed lytic and sclerotic skeletal lesions were also showing intermediate to high PSMA expression (miPSMA expression score of 2-3, with the highest score of 3), with overall stage of miT4N2 (bilateral II, EI, OB, PS, OP), M1a (bilateral CI, RP, OE) b (diss), and lowest PSMA expression score 1 and highest 3, according to PROMISE V2 criteria (Figure 3).

The patient then underwent left-sided percutaneous nephrostomy tube insertion, after which his creatinine levels came down to 1.7 mg/dL. Further management was discussed in a multidisciplinary tumor board, and then, the treatment options, either surgery or hormonal therapy, were offered to the patient. In view of his socioeconomic status, the patient opted for surgery for the management of extensive prostatic cancer disease. The proctitis was managed conservatively.

## Discussion

PSMA is a transmembrane protein that is overexpressed in adenocarcinoma cells than in normal prostatic parenchyma [4]. Although it is termed specific, PSMA expression is also seen in certain non-prostatic tumors and inflammatory conditions, primarily along endothelial cells of neovasculature. Increased vascularity and macrophage folate receptor expression are possible reasons for increased PSMA ligand uptake in infection and inflammatory conditions [4]. Few case reports have demonstrated intermediate to high PSMA expression in synchronous rectal primary along with prostate cancer uptake [4-7]. Rectal lesion biopsy for this patient turned out to be inflammatory with normal serum CEA levels in spite of strong clinical and radiological suspicion of synchronous second primary. Low to intermediate PSMA expression in the rectal lesion in this case was comparable to other case series and case reports, which demonstrated similar PSMA expression in inflammatory bowel diseases [8,9]. Hence, the PSMA expression score of rectal lesions might be able to differentiate between benign inflammation and malignant pathology in suspected non-prostatic synchronous rectal primary cases.

It is known that the intensity of PSMA expression is upregulated in higher ISUP grade prostatic adenocarcinomas and in significant metastatic diseases [3]. Although in this patient, the prostatic lesion was infiltrating bilateral seminal vesicles and the base of the urinary bladder and had a higher ISUP group grade of 5, the PSMA expression score of the primary lesion was of low to intermediate grade. Thus, the PRIMARY score of the prostatic lesion is 2 and is negative according to PROMISE V2 criteria, as it only considers the intensity of PSMA expression, as well as intraprostatic uptake pattern and location for scoring [3]. Very few studies have demonstrated a small subset of high-grade prostatic primary lesions with low PSMA expression, which needs to be addressed in future revisions of the PROMISE criteria. This might be due to tumor dedifferentiation and modifications in the *FOLH1* gene encoding PSMA protein, and may represent poor prognosis [10]. F-18 fluorodeoxyglucose (FDG) PET might help in understanding tumor heterogeneity in these types of prostatic adenocarcinoma and help in guiding further therapy.

## Conclusions

Low-grade PSMA expression in high ISUP grade prostatic primary with significant metastases can occur. Hence, it is important to integrate PSMA PET scoring systems with histopathological results for final clinical decision-making. miTNM staging, where miT depends on uni- or multifocality and the extent of involvement of neighboring structures, will better describe prostatic primary lesion with low-grade PSMA expression than the proposed PRIMARY score in PROMISE V2 criteria. PSMA expression score might help in characterizing synchronous rectal lesions in patients with prostate adenocarcinoma.
